# The endocytic recycling regulator EHD1 is essential for spermatogenesis and male fertility in mice

**DOI:** 10.1186/1471-213X-10-37

**Published:** 2010-04-02

**Authors:** Mark A Rainey, Manju George, GuoGuang Ying, Reiko Akakura, Daniel J Burgess, Ed Siefker, Tom Bargar, Lynn Doglio, Susan E Crawford, Gordon L Todd, Venkatesh Govindarajan, Rex A Hess, Vimla Band, Mayumi Naramura, Hamid Band

**Affiliations:** 1 Eppley Institute for Research in Cancer and Allied Diseases, UNMC-Eppley Cancer Center, University of Nebraska Medical Center, Omaha, Nebraska, USA; 2 Laboratory of Molecular Oncology, Tianjin Medical University Cancer Institute and Hospital, Tianjin, PR China; 3 Department of Biochemistry and Molecular Biology, UNDNJ-New Jersey Medical School, Newark, New Jersey, USA; 4 Department of Surgery, Creighton University, Omaha, Nebraska, USA; 5 Department of Genetics, Cell Biology and Anatomy, College of Medicine, University of Nebraska Medical Center, Omaha, Nebraska, USA; 6 Department of Medicine, Northwestern University Feinberg School of Medicine, Chicago, Illinois, USA; 7 Department of Pathology, Northwestern University Feinberg School of Medicine, Chicago, Illinois, USA; 8 Department of Veterinary Bioscience, University of Illinois at Urbana-Champaign, Urbana, Illinois, USA; 9 Department of Biochemistry and Molecular Biology, College of Medicine, University of Nebraska Medical Center, Omaha, Nebraska, USA

## Abstract

**Background:**

The C-terminal Eps15 homology domain-containing protein 1 (EHD1) is ubiquitously expressed and regulates the endocytic trafficking and recycling of membrane components and several transmembrane receptors. To elucidate the function of EHD1 in mammalian development, we generated *Ehd1*^-/- ^mice using a Cre/*loxP *system.

**Results:**

Both male and female *Ehd1*^-/- ^mice survived at sub-Mendelian ratios. A proportion of *Ehd1*^-/- ^mice were viable and showed smaller size at birth, which continued into adulthood. *Ehd1*^-/- ^adult males were infertile and displayed decreased testis size, whereas *Ehd1*^-/- ^females were fertile. *In situ *hybridization and immunohistochemistry of developing wildtype mouse testes revealed EHD1 expression in most cells of the seminiferous epithelia. Histopathology revealed abnormal spermatogenesis in the seminiferous tubules and the absence of mature spermatozoa in the epididymides of *Ehd1*^-/- ^males. Seminiferous tubules showed disruption of the normal spermatogenic cycle with abnormal acrosomal development on round spermatids, clumping of acrosomes, misaligned spermatids and the absence of normal elongated spermatids in *Ehd1*^-/- ^males. Light and electron microscopy analyses indicated that elongated spermatids were abnormally phagocytosed by Sertoli cells in *Ehd1*^-/- ^mice.

**Conclusions:**

Contrary to a previous report, these results demonstrate an important role for EHD1 in pre- and post-natal development with a specific role in spermatogenesis.

## Background

The C-terminal Eps15 homology domain-containing (EHD) proteins regulate endocytic recycling of membrane and associated cell surface receptors [1]. The founding EHD family member, the single *C. elegans *ortholog RME-1 (Receptor-Mediated Endocytosis-1), was identified in a screen for mutants defective in yolk protein endocytosis, and is required for yolk receptor and basolateral fluid recycling in the worm [2]. Mutations of the single *Drosophila *EHD protein ortholog Past1 decreased fertility and germline development in the fly [3]. Mammals express four highly homologous EHD proteins (EHD1-4) each containing an N-terminal ATPase domain [4,5], a central coiled-coil region that facilitates homo- and hetero-oligomerization [6-8], and a single C-terminal Eps15 homology (EH) domain that mediates interactions with proteins containing Asn-Pro-Phe motifs [9,10]. Ectopic expression of each human EHD protein in *C. elegans rme-1 *mutants rescued the basolateral recycling defect indicating a basic functional similarity of human EHD proteins and RME-1 [7]. However, the presence of four EHD proteins in mammals suggests tissue-specific and/or non-redundant roles of individual family members.

The sorting of endocytosed receptors determines whether they are recycled to the cell surface or degraded in the lysosomes. Receptors destined for recycling are trafficked through either a fast recycling pathway from the early endosomes (EEs) or through a slow recycling pathway through the endocytic recycling compartment (ERC) [11-13]. EHD proteins appear to regulate critical nodes in the endocytic sorting/recycling process [14]. Several lines of evidence suggest that EHD1 regulates the rate of ERC to cell surface trafficking in the slow recycling pathway. Overexpression of a dominant-negative mouse EHD1 mutant (G429R) disrupted the morphology of the ERC and slowed the exit of transferrin from the ERC [15]. Knock-down of EHD1 using siRNA delayed the release of transferrin and decreased surface levels of β1 integrin due to reduced recycling from the ERC [16]. Co-overexpression studies demonstrated that recycling of the major histocompatibility complex (MHC) class I molecule H-2D^d ^from an intracellular compartment to the cell surface was increased with EHD1 overexpression [17] and EHD1 depletion led to retention of MHC class I in a compact pericentriolar compartment reminiscent of the ERC [18]. EHD1 also maintains the perinuclear localization of glucose transporter 4 in cultured adipocytes [19] and positively regulates the kinetics of endosome-to-Golgi retrieval of the cation-independent mannose-6-phosphate receptor [20].

We previously suggested that EHD4 regulates the EE to ERC transport of transferrin based on siRNA-mediated depletion of EHD4 [7]. In support of this hypothesis, EHD4 depletion led to retention of recycling-destined transferrin or MHC class I and lysosome-destined low-density lipoproteins in enlarged EEs, suggesting that EHD4 regulates the rate of exit of trafficking receptors from the EEs towards both the ERC and lysosomal degradation routes [8]. In neuronal cells, EHD4 (also known as Pincher) also mediates formation of clathrin-independent macroendosomes of TrkA and TrkB receptor tyrosine kinases [21].

EHD3 has been ascribed two distinct roles in regulating the exit of traffic from the EEs to both the ERC [22] and the Golgi [23]. EHD3 depletion led to fragmentation of the Golgi [23]. Although less studied, EHD2 has been ascribed a role in endocytosis [24], nucleotide-dependent membrane remodeling [5] and fusion of myoblasts [25].

To date, studies of the EHD protein family have largely focused on their role in trafficking transferrin and receptors using *in vitro *assays. In contrast to *in vitro *studies implicating mammalian EHD proteins in the regulation of endocytic recycling, the only evidence for their *in vivo *roles is by analogy to RME-1 in *C. elegans *and Past1 in *Drosophila*. Direct evidence for an *in vivo *function of EHD proteins in mammalian systems is presently lacking. Analyses of the expression of EHD paralogs in different mouse tissues are consistent with the likelihood that different EHD proteins may have tissue-specific as well as more redundant roles [7]. Early studies highlighted the relatively high *Ehd1 *mRNA and protein expression in mouse testis (human EHD1 is also known as Testilin [GenBank: AF099011]), kidney, heart, intestine and brain [9]. In the same study, immunohistochemistry revealed EHD1 protein expression in elongated spermatids in the testis, adipocytes, lung, heart and specific retinal layers in mice [9]. EHD1 has also been found in exosome-like vesicles purified from the cauda epididymal fluid of rams [26]. Contrary to expectations based on a relatively high expression in certain organs, targeted deletion of the EHD1 C-terminal region in mice did not produce an overt phenotype [27]. Given the plethora of *in vitro *cell biological studies supporting a role for mammalian EHD1, we used a different targeting strategy to generate an *Ehd1 *knockout mouse that completely lacks EHD1 expression and assessed whether the loss of EHD1 had any demonstrable impact on adult organ function. In contrast to previous results [27], we report that *Ehd1*-null mice survive at sub-Mendelian ratios in several mouse strains, display reduced growth as compared to wildtype (WT) mice and *Ehd1*^-/- ^males are infertile. We conclude that the endocytic recycling regulator EHD1 plays an important role in mouse development and is essential for male fertility. To our knowledge, this is the first knockout mouse model of male infertility due to the loss of a single protein implicated in endocytic recycling.

## Results

### Generation of EHD1-deficient mice

**Figure 1:**
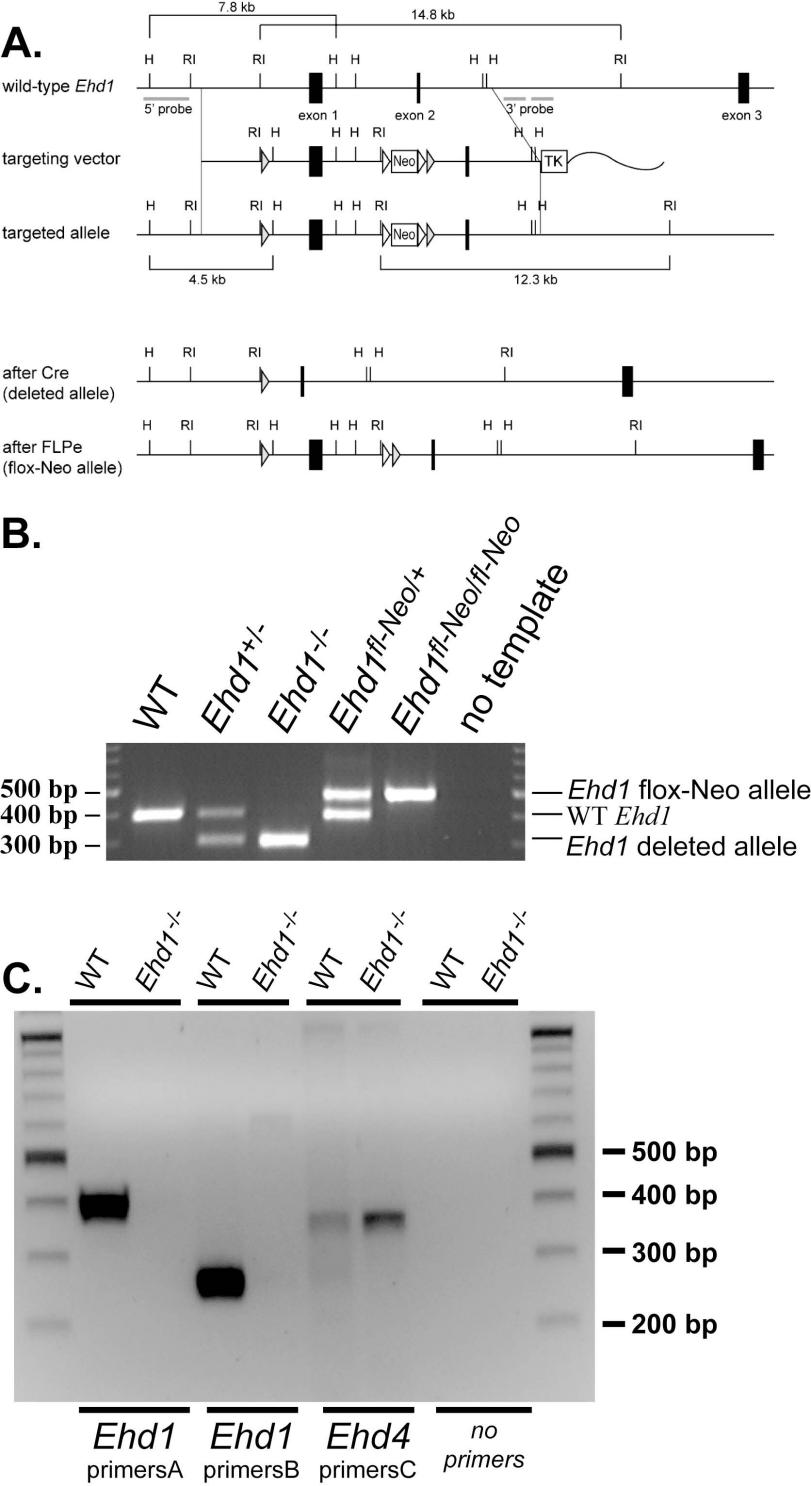
**Generation of *Ehd1*^-/- ^mice using Cre/*loxP*-mediated genetic recombineering**. (A) A partial restriction map of the *Ehd1 *locus, the targeting vector and the mutated *Ehd1 *loci. The first exon was deleted by Cre/*loxP*-mediated recombination. Black rectangles represent exons, grey and white triangles represent *loxP *and *FRT *sequences, respectively. H, *Hind*III; RI, *Eco*RI. (B) DNA was prepared from mouse tails for genotyping by PCR to amplify the WT *Ehd1 *allele, the deleted allele and/or the floxed allele. The lane labeled "no template" indicates a negative control in the absence of DNA. (C) RT-PCR analysis was carried out using cDNA generated from mouse testes and primers specific for *Ehd1 *and *Ehd4*. The primers are described in Methods.

EHD1-deficient mice were generated using a recombineering strategy as described in Methods (Figure 1A). PCR analysis of tail DNA confirmed the *Ehd1 *gene was correctly targeted in heterozygous deleted (*Ehd1*^+/-^), homozygous deleted (*Ehd1*^-/-^), heterozygous floxed (*Ehd1*^*fl-Neo*/+^), and homozygous floxed (*Ehd1*^*fl-Neo/fl-Neo*^) mice (Figure 1B). RT-PCR also confirmed the absence of *Ehd1 *mRNA in the testis of *Ehd1*^-/- ^male mice (Figure 1C).

**Figure 2:**
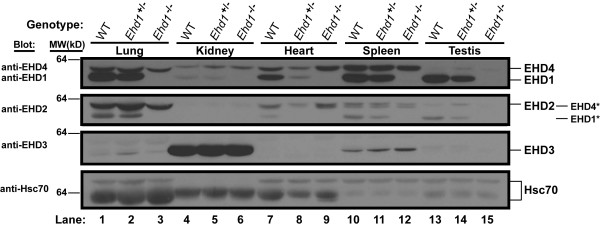
**EHD protein expression in adult WT, *Ehd1*^+/- ^and *Ehd1*^-/- ^male mice**. Aliquots of 100 μg tissue lysates derived from seven month old male mice were separated using 7.5% SDS-PAGE and Western blots were performed using antisera raised against EHD proteins as described in Methods. The * denotes bands that bled through from the previous blot following stripping. Differential mobility of Hsc70 may represent tissue specific isoforms. Relative molecular weight (MW) markers are indicated in kiloDaltons (kD). Hsc70 served as a loading control.

Previously, we showed that EHD proteins were expressed in several mouse organs in both male and female mice [7]. Western blots performed on lysates of mouse organs obtained from WT, *Ehd1*^+/- ^and *Ehd1*^-/- ^mice confirmed that disruption of *Ehd1 *led to a loss of EHD1 protein expression in *Ehd1*^-/- ^male (Figure 2) as well as female mice (data not shown). Intermediate levels of EHD1 were seen in the lung, kidney, heart, spleen, and testis of *Ehd1*^+/- ^mice when compared to WT and *Ehd1*^-/- ^mice (Figure 2). These results demonstrated that the targeting strategy led to complete loss of EHD1 expression in *Ehd1*^-/- ^mouse tissues.

### Deletion of *Ehd1 *in different mouse strains results in partial lethality

Crosses of *Ehd1*^+/- ^mice on a 129;B6 mixed background did not produce the expected Mendelian ratio of *Ehd1*^-/- ^mice (8% instead of the expected 25% were *Ehd1*^-/- ^at post-natal days 10-12) (Table 1). These results indicated that loss of EHD1 was partially lethal. Similar results were seen after seven backcrosses (N7) to the FVB/NJ strain (11% instead of the expected 25% were *Ehd1*^-/-^; n = 106 mice). The 129;B6 mixed strain was used in further analyses unless specified.

**Table 1:** Genotypes of pups obtained from *Ehd1 *mutant mouse breeding schemes

	Female	Male	WT pups	***Ehd1***^**+/- **^**pups**	***Ehd1***^**-/- **^**pups**	Total
N* = 15	*Ehd1*^+/-^	*Ehd1*^+/-^	82 (33%)	148 (59%)	19 (8%)	249

N* = 9	*Ehd1*^-/-^	*Ehd1*^+/-^	0	44 (71%)	18 (29%)	62

N = 8	WT	*Ehd1*^-/-^	0	0	0	0

N denotes the number of breeding pairs; * - some breeding pairs produced multiple litters. % was calculated for each genotype based on total pups for each breeding scheme. *Ehd1*^-/- ^males were bred for two months with two females each.

The progeny from crosses of *Ehd1*^+/- ^mice were 49% female and 51% male with twice as many *Ehd1*^+/- ^as compared to WT mice, indicating normal gender ratios and a lack of lethality when one copy of *Ehd1 *was present. A separate study was conducted where the genotype of pups that died from unknown causes between post-natal days 0 and 2 were examined. Interestingly, 24 of 48 pups (50%) were *Ehd1*^-/- ^mice, indicating a disproportionately higher frequency (expected ~25%) of death among *Ehd1*^-/- ^mice at or near birth.

### *Ehd1*^-/- ^mice are smaller than WT mice and display developmental defects

**Figure 3:**
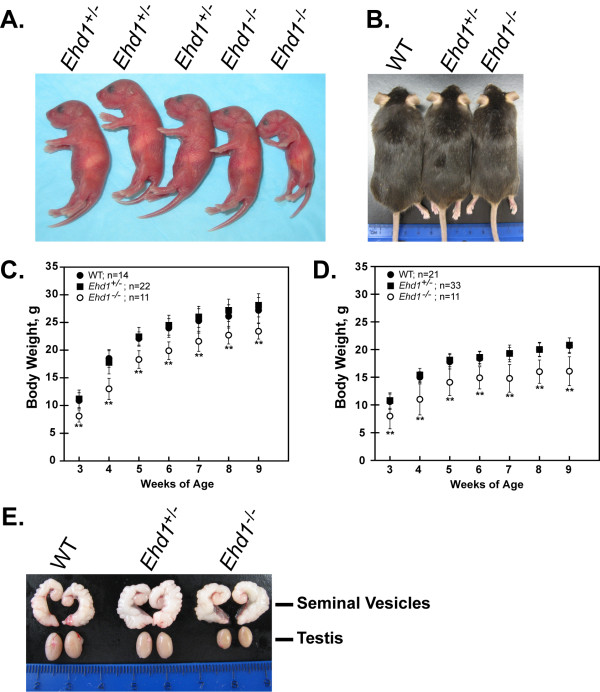
***Ehd1*^-/- ^mice are smaller than littermate controls and adult *Ehd1*^-/- ^males exhibit small testis**. (A) Newborn pups and (B) seven month old male mice were photographed to show the size of the *Ehd1*^-/- ^mice as compared to littermate controls. (C) Quantitative growth curves of male and (D) female littermate control mice (n value shown for each). (E) Seminal vesicles and testis were dissected from mice pictured in (B). Error bars represent standard deviation from the mean. ** indicates statistically significant using a two-sample t-test with a two-tailed analysis (p < 0.05).

*Ehd1*^-/- ^mice that survived early neonatal lethality were smaller than WT and *Ehd1*^+/- ^littermates from birth (Figure 3A) to adulthood (Figure 3B). Both male and female mice showed lower weights as compared to controls (Figure 3C-D). In several cases, *Ehd1*^-/- ^females displayed malocclusion (4/18, 22%) which required bi-weekly incisor trimming into adulthood to prevent death. A few animals were euthanized due to abnormally small size and malnutrition at age 3-4 weeks independent of incisor problems and several others perished around this time due to unknown causes. A substantial proportion of the surviving *Ehd1*^-/- ^animals displayed gross ocular defects (~55% of eyes; n = 39 animals) including anophthalmia (rare), microphthalmia (severe cases exhibited closed eyelids), and congenital central cataracts. The nature of eye developmental defects in *Ehd1*^-/- ^mice is being pursued separately.

### *Ehd1*^-/- ^male mice are infertile

Despite our repeated attempts to mate *Ehd1*^-/- ^mice, no progeny were generated indicating the lack of fertility of either one or both genders. Breeding *Ehd1*^+/- ^males with *Ehd1*^-/- ^females gave rise to healthy pups (Table 1); only 29% (instead of 50% expected) of the mice that survived to weaning age were *Ehd1*^-/-^. For unknown reasons, the percentage of *Ehd1*^-/- ^mice surviving to weaning age compared to Mendelian predictions were higher when raised by an *Ehd1*^-/- ^dam versus an *Ehd1*^+/- ^dam. These results further documented that *Ehd1*^-/- ^females were fertile and the partial lethality in *Ehd1*-null mice (currently under investigation).

To test if *Ehd1*^-/- ^males were fertile, 8-week old males were housed with two virgin adult females each. Despite normal mating behavior, as determined by their ability to mount females and give rise to a copulatory plug, no *Ehd1*^-/- ^male mice were capable of siring offspring, indicating that *Ehd1*^-/- ^males were infertile (Table 1). Females used in these experiments were proven competent at being impregnated by other fertile males after initial breeding with *Ehd1*^-/- ^males. The ability of eight *Ehd1*^*fl-Neo/fl-Neo *^breeding pairs to successfully produce progeny provided evidence that the presence of *loxP *sites in the targeted *Ehd1 *gene did not cause defects in fertility or overall survival by influencing an untargeted gene.

Previously, a C-terminal deletion of EHD1 in mice was shown to have no effects in viability, growth or fertility in 129/SvEv or Swiss Webster strains [27]. To determine whether fertility defects in the *Ehd1*^-/- ^male mice were influenced by genetic background, we crossed *Ehd1*^+/- ^mice two times into the FVB/NJ strain (N2) and then generated *Ehd1*^-/- ^mice. The resulting *Ehd1*^-/- ^male mice were infertile while *Ehd1*^-/- ^female mice were fertile (n = 8). Further backcrossing revealed that *Ehd1*^-/- ^male FVB/NJ strain (N7) mice were also infertile (n = 4), indicating that loss of EHD1 leads to complete infertility in male mice irrespective of strain.

### Adult *Ehd1*^-/- ^male mice exhibit small testes

The raw weights of testes, spleen and kidneys of *Ehd1*^-/- ^male mice at post-natal day 10 and 30 were not statistically different from WT mice (Table 2). However, from day 42, the testes in *Ehd1*^-/- ^mice were smaller than that of WT mice indicating the first delay in testes development as determined by weight (Table 2, Figure 3E). Interestingly, the androgen-dependent seminal vesicles were comparable in size between WT, *Ehd1*^+/- ^and *Ehd1*^-/- ^mice (Table 2, Figure 3E) suggesting that hormone levels were unaffected. Serum testosterone levels of mice were variable; however, levels in *Ehd1*^-/- ^mice were within a range comparable to those of WT and *Ehd1*^+/- ^adult mice (824.5 ± 1364.0 ng/dL for WT [n = 3], 646.1 ± 859.4 ng/dL for *Ehd1*^+/- ^[n = 2] and 445.8 ± 511.4 for *Ehd1*^-/- ^[n = 11], ages 9-69 weeks, p > 0.05). The small testis size phenotype was similar in N2 and N7 FVB/NJ mice (data not shown).

**Table 2:** Uncorrected organ weights and seminiferous tubule widths of WT and *Ehd1*^-/- ^male mice

Age	Genotype	Testes, mg	**Seminiferous Tubule Width, mm × 10**^**-1**^	Spleen, mg	Kidneys, mg
**Day 10**	**WT**	8.9 ± 1.6	1.3 ± 0.1	21.8 ± 2.2	57.6 ± 9.3

**Day 10**	***Ehd1***^-/-^	7.0 ± 1.0	1.2 ± 0.1	23.2 ± 8.5	46.5 ± 13.5

**Day 30**	**WT**	98.2 ± 10.7	2.9 ± 0.3	78.9 ± 13.9	238.2 ± 16.4

**Day 30**	***Ehd1***^-/-^	90.5 ± 8.1	3.1 ± 0.2	90.0 ± 8.0	242.2 ± 39.7

**Day 42**	**WT**	172.7 ± 5.9*	3.6 ± 0.4	83.1 ± 10.2	358 ± 43.5

**Day 42**	***Ehd1***^-/-^	127.7 ± 24.8*	3.6 ± 0.4	75.1 ± 6.7	324.7 ± 3.2

**Weeks 9-11**	**WT**	197.0 ± 17.5**	n/a	63.7 ± 10.6	392.7 ± 30.6

**Weeks 9-11**	***Ehd1***^-/-^	106.3 ± 17.1**	n/a	69.0 ± 13.1	362 ± 15.9

**Weeks 19-21**	**WT**	227.0 ± 2.0**	n/a	81.2 ± 7.0	442.4 ± 78.2

**Weeks 19-21**	***Ehd1***^-/-^	136.7 ± 14.4**	n/a	65.3 ± 11.6	341.3 ± 35.9

**Weeks 61-69**	**WT**	185.4 ± 31.3*	n/a	70.2 ± 7.6	502.2 ± 38.2

**Weeks 61-69**	***Ehd1***^-/-^	93.5 ± 6.9*	n/a	60.2 ± 11.5	468.7 ± 136.7

Organs were dissected from euthanized mice and weighed. The testes weight represents both testis dissected from the scrotal sac, the seminiferous tubule widths (n ≥ 20) were measured using ImageJ software http://rsbweb.nih.gov/ij/ to measure the smallest width of circular seminiferous tubules across the lumen from light microscopy photographs of PAS-stained Bouin's-fixed testis sections. The kidney weight represents both kidneys (n > 3 for each measurement). The uncorrected seminal vesicle weights of 61-69 week old mice: WT = 569.9 ± 184.5 mg and *Ehd1*^-/- ^= 411.1 ± 89.3 mg (p > 0.05). Statistical analysis comparing the raw data of each age-matched values between WT and *Ehd1*^-/- ^mice was determined using a two-sample t-test (* indicates p < 0.05 using one-tailed analysis, ** indicates p < 0.05 using two-tailed analysis).

### EHD1 expression in post-natal mouse testis development

**Figure 4:**
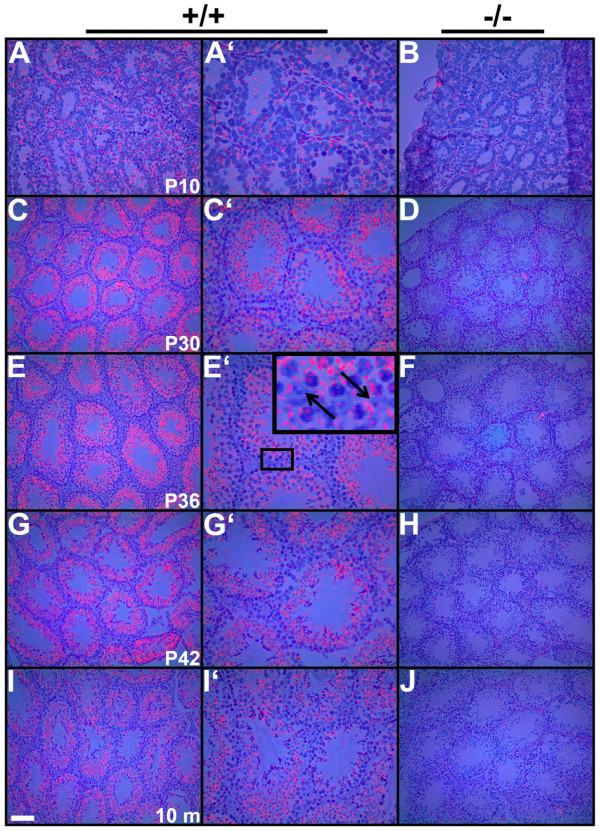
***Ehd1 mRNA expression *in post-natal mouse testis development**. *In situ *hybridizations were performed as described in Methods on WT (+/+) and *Ehd1*^-/- ^(-/-) testis sections prepared from post-natal day 10, 30, 36, 42 (P10-P42) and 10 month old (10 m) mice. *Ehd1 *mRNA expression can be seen as red in dark-field images overlaid on bright-field images. Panels A'-I' are higher magnifications of panels A-I, respectively. The inset within E' is an enlarged micrograph of the box in E'; arrows denote Sertoli cell nuclei. The scale bar in panel I is 100 μm for B-J, 50 μm for A, C'-I' and 25 μm for A'.

To assess the *Ehd1 *mRNA expression, *in situ *hybridizations were carried out in developing mouse testes. *Ehd1 *mRNA was expressed in most cells of the seminiferous epithelia (Figure 4) including Sertoli cells (Figure 4, E' inset).

**Figure 5:**
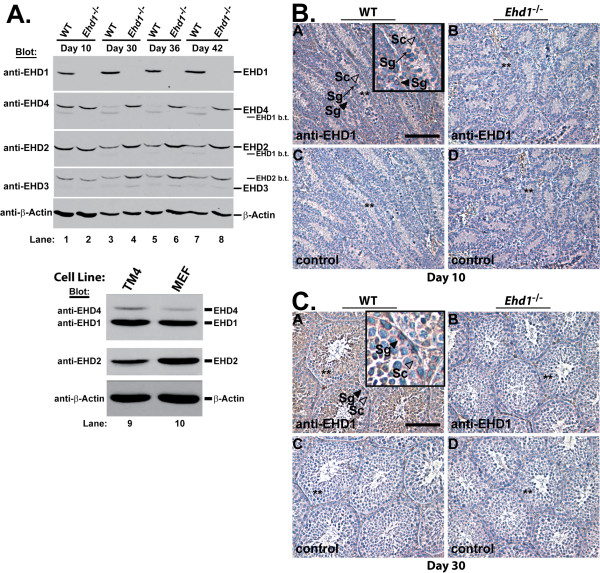
**EHD protein expression and EHD1 localization during mouse testis development**. (A, upper panel) Aliquots of 50 μg testis lysates from post-natal day 10-42 mice were separated using 8% SDS-PAGE and a Western blot was performed using affinity purified antibodies raised against EHD1 (described in Methods), followed by serial reprobing with antisera raised against EHD proteins as described previously [7]. β-Actin served as a loading control. The * denotes bands that bled through from the previous blot. (A, lower panel) Aliquots of 20 μg immortalized mouse TM4 Sertoli cell and mouse embryonic fibroblast (MEF, *Ehd1*^*fl-Neo/fl-Neo*^) lysates were treated similarly except the membrane was probed with antisera that recognize EHD1 and EHD4 followed by EHD2. (B, C) Immunohistochemistry was carried out to determine EHD1 localization in day 10 and day 30 formalin-fixed testis sections from WT and *Ehd1*^-/- ^mice. EHD1 expression can be seen as brown staining; nuclei are counter-stained with hematoxylin (blue). Panels A and B contained affinity purified anti-EHD1 primary antibodies while panels C and D lacked primary antibodies (control). Insets in panel A are enlarged micrographs of the highlighted cells. Note: similar seminiferous tubules from adjacent sections can be seen in A and C as well as B and D; denoted by asterisks (**). Sc - Sertoli cell, Sg - spermatogonia. Scale bar = 100 μm.

To assess the EHD protein expression at early stages of testis development, a Western blot was performed (Figure 5A, upper panel). EHD1, EHD2 and EHD4 were expressed in WT testis at days 10-42 while EHD3 levels were relatively low. Interestingly, *Ehd1*^-/- ^testes displayed an increase in EHD2, EHD3 and EHD4 expression at day 30, 36 and 42. EHD1, EHD2 and EHD4 were also expressed in an immortalized mouse Sertoli cell line (TM4) as analyzed by Western blot (Figure 5A, lower panel).

To determine EHD1 localization within the testis, immunohistochemistry was performed and revealed EHD1 expression in most cells of the seminiferous epithelium. At post-natal day 10, EHD1 was expressed in the cytoplasm of both spermatogonia (filled arrowheads) and Sertoli cells (open arrow-heads) at the basement membrane with higher signals near the lateral and apical surfaces of these cells (Figure 5B, panel A). EHD1 expression was also seen around the nucleus of spermatogonia (arrow) towards the lumen of seminiferous tubules (Figure 5B, panel A). As expected, EHD1 expression was absent in *Ehd1*^-/- ^testis (Figure 5B-C, panel B). At day 30, EHD1 was localized in the cytoplasm of Sertoli cells and spermatogonia near the base of seminiferous tubules. In addition, EHD1 was expressed in pachytene spermatocytes, and round and elongated spermatids (Figure 5C, panel A). Similar patterns were observed in adult WT testis (not shown).

### *Ehd1*^-/- ^mice show a range of abnormalities in spermatogenesis

During spermatogenesis, spermatogonia undergo mitotic divisions and differentiate into spermatocytes. Spermatocytes undergo two meiotic divisions, differentiate to round spermatids that later form elongated spermatids and are released from Sertoli cells during spermiation. Progression of spermatogenesis is described using histology of seminiferous tubule cross-sections in *stages *(I-XII) that define the morphological development of germ cells as a group [28,29]. The morphology of an individual spermatid in spermiogenesis is described as a *step *that is most easily followed by Periodic Acid-Schiff (PAS)-stained acrosome formation and shape, or less easily by assessing chromatin condensation and spermatid head shape [28]. In order to discern initial lesions in spermatogenesis, we carried out histological analyses of testes in 10, 30, and 42 day old mice. At each age, the average width of seminiferous tubules was comparable between WT and *Ehd1*^-/- ^mice indicating that lumen formation by the seminiferous epithelia was unaffected in the absence of EHD1 (Table 2).

**Figure 6:**
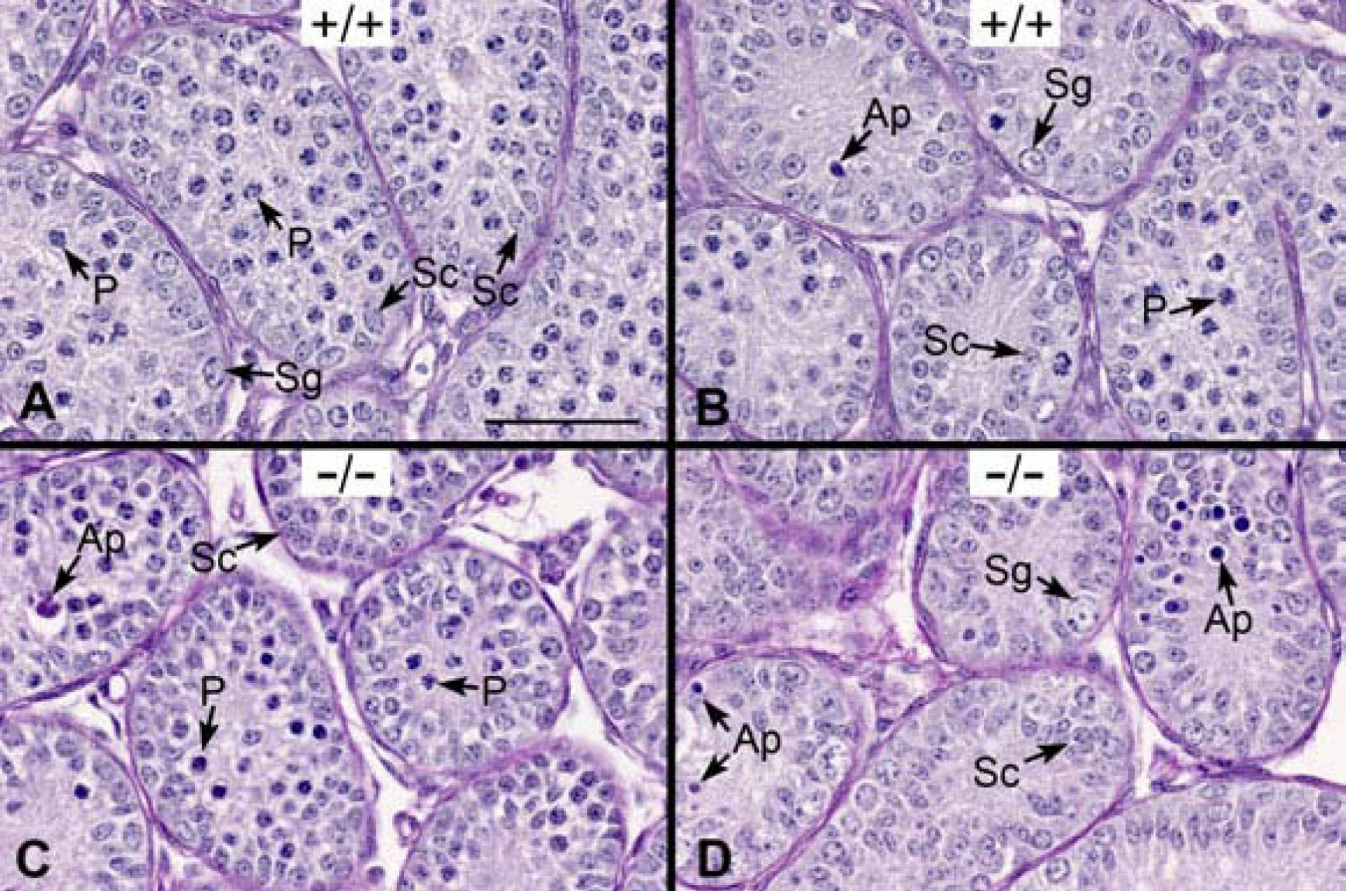
**Post-natal day 10 tubule cross-sections of *Ehd1*^-/- ^male mouse testes show no major lesions**. Day 10 testes were Bouin's-fixed, PAS-stained and hematoxylin-counter-stained to visualize the glycoproteins/acrosomes (pink) and nuclei (blue) and analyzed by light microscopy using a 40× objective lens. Stages are labeled with Roman numerals. (A, B) The seminiferous tubules of WT (+/+) mice exhibit Sertoli cell nuclei (Sc) near the basement membrane or toward the lumen, large spermatogonia (Sg) near the basement membrane, pachytene spermatocytes (P) and occasional apoptotic-like (Ap) nuclei near the lumen. (C, D) The seminiferous tubules of *Ehd1*^-/- ^(-/-) mice are shown for comparison. Scale bar in A = 50 μm for A-D.

At post-natal day 10, the WT seminiferous tubules predominantly contained Sertoli cells and some pachytene spermatocytes, the most advanced germ cell type seen at this age (Figure 6A). Spermatogonia were present near the basement membrane and few apoptotic features were seen (Figure 6B) [30]. *Ehd1*^-/- ^seminiferous tubules were similar in appearance, also displaying some apoptotic features in the lumen and near the basement membrane (Figure 6C-D). There appeared to be a delay in the normal maturation of spermatogonia and pachytene spermatocytes in *Ehd1*^-/- ^as compared to WT mice as analyzed by chromatin condensation and cell size. In addition, some *Ehd1*^-/- ^seminiferous tubules contained a greater number of apoptotic-like dense bodies than WT. However, no major lesions were detected in the seminiferous tubules of *Ehd1*^-/- ^mice at day 10.

**Figure 7:**
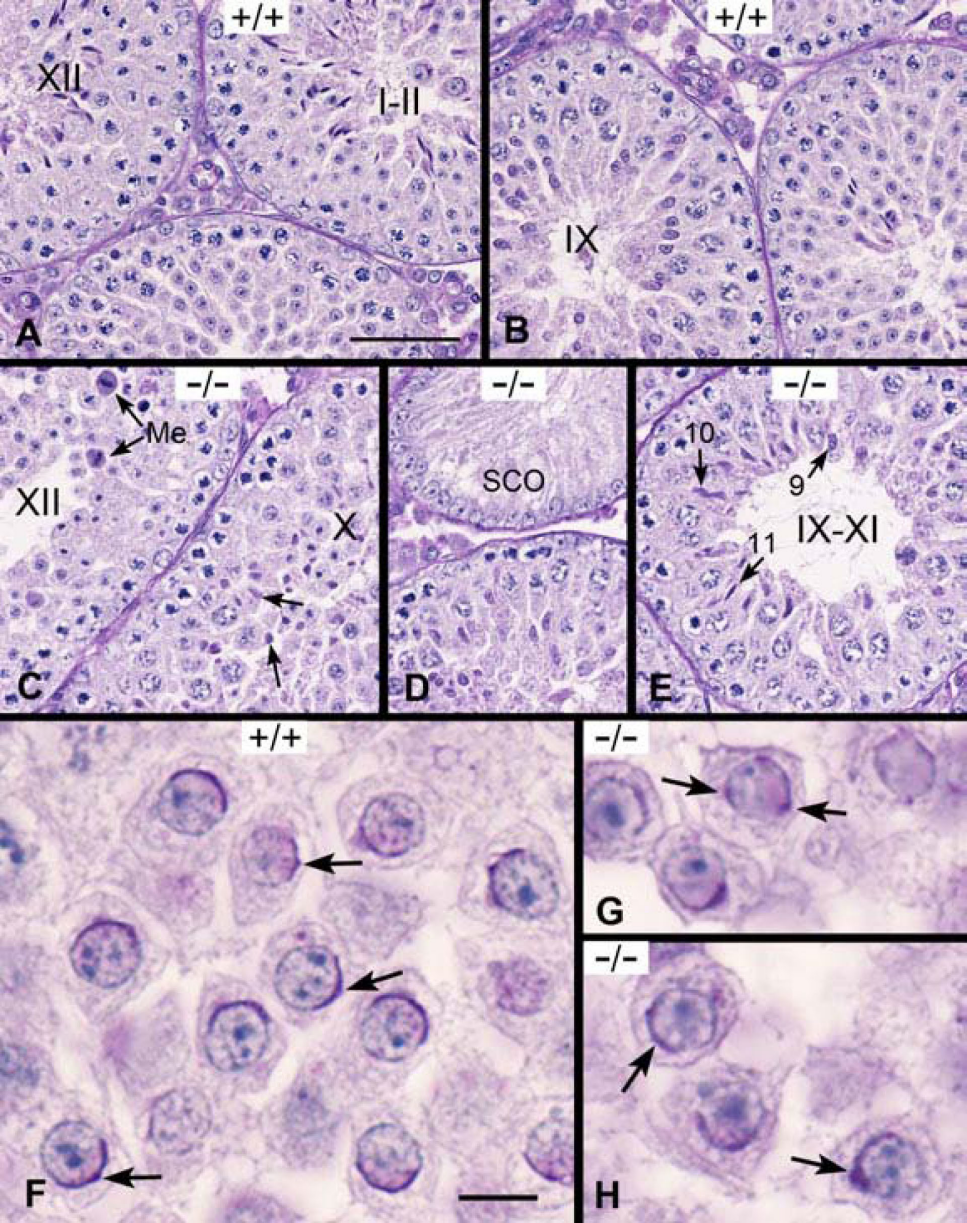
**Abnormal acrosome and spermatid development in adolescent *Ehd1*^-/- ^male mice**. Day 30 testes were Bouin's-fixed, PAS-stained and hematoxylin-counter-stained to visualize the glycoproteins/acrosomes (pink) and nuclei (blue) and analyzed by light microscopy using a 40× objective lens (A-E) or 60× objective lens under oil immersion (F-H). Stages are labeled with Roman numerals. (A) WT (+/+) stage XII seminiferous tubules with step 12 elongated spermatids and spermatocytes in meiosis I and II. (B) WT stage IX seminiferous tubules with step 9 spermatids. (C) *Ehd1*^-/- ^(-/-) stage XII seminiferous tubules display abnormal meiotic figures (Me). Stage X shows a mixture of spermatid steps with abnormal orientation, shape and chromatin condensation (arrows). (D) An *Ehd1*^-/- ^seminiferous tubule exhibiting a Sertoli cell only (SCO) phenotype and a (E) stage IX-XI seminiferous tubule containing step 9, 10 and 11 elongated spermatids (arrows). (F) Stage VII WT round spermatids with PAS-positive acrosomal caps on developing step 7 round spermatids (arrows). (G-H) *Ehd1*^-/- ^step 7 round spermatids display abnormal acrosomal caps (arrows), while others show abnormal displacement of the acrosomal granule, asymmetric formations and punctuate appearances. Scale bar in A = 50 μm for A-E. Scale bar in F = 10 μm for F-H.

At day 30, normal spermatogenesis was apparent in WT mice with well-organized germ cells typical of the first wave of spermatogenesis. In general, Sertoli cells were near the basement membrane while elongated spermatids lined the lumen (Figure 7A, stage I-II) and normal meiosis was observed in spermatocytes (Figure 7A, stage XII). Round spermatids displayed a round/ovoid appearance until stage IX when the spermatid head formed a dorsal and ventral surface with the acrosome primarily on the dorsal surface of step 9 spermatids (Figure 7B). In contrast, *Ehd1*^-/- ^seminiferous tubules showed abnormal cells in meiosis (Figure 7C, stage XII) and elongated spermatids that displayed abnormal orientation, shape and chromatin condensation (Figure 7C, stage X). Some *Ehd1*^-/- ^seminiferous tubules displayed a Sertoli cell only phenotype not seen in the WT (Figure 7D) indicating a complete lack of germ cells. Interestingly, a delay in the maturation of elongated spermatids was observed with a mixture of spermatids (step 9, 10, and 11) present in a seminiferous tubule cross-section (Figure 7E). In WT mice, the PAS-positive acrosomal cap of round spermatids covered more than one third of the nucleus at stage VII with a central acrosomal granule (Figure 7F). However, in *Ehd1*^-/- ^mice, the acrosomal caps appeared abnormal with asymmetric formations (Figure 7G) and punctate appearances (Figure 7H). Neither the *Ehd1*^-/- ^nor the WT epididymides contained spermatozoa at day 30, confirming that these animals were in the initial waves of spermatogenesis. Since round spermatids form prior to day 30 (days 20-25), there may be lesions that were not elucidated in the current study.

**Figure 8:**
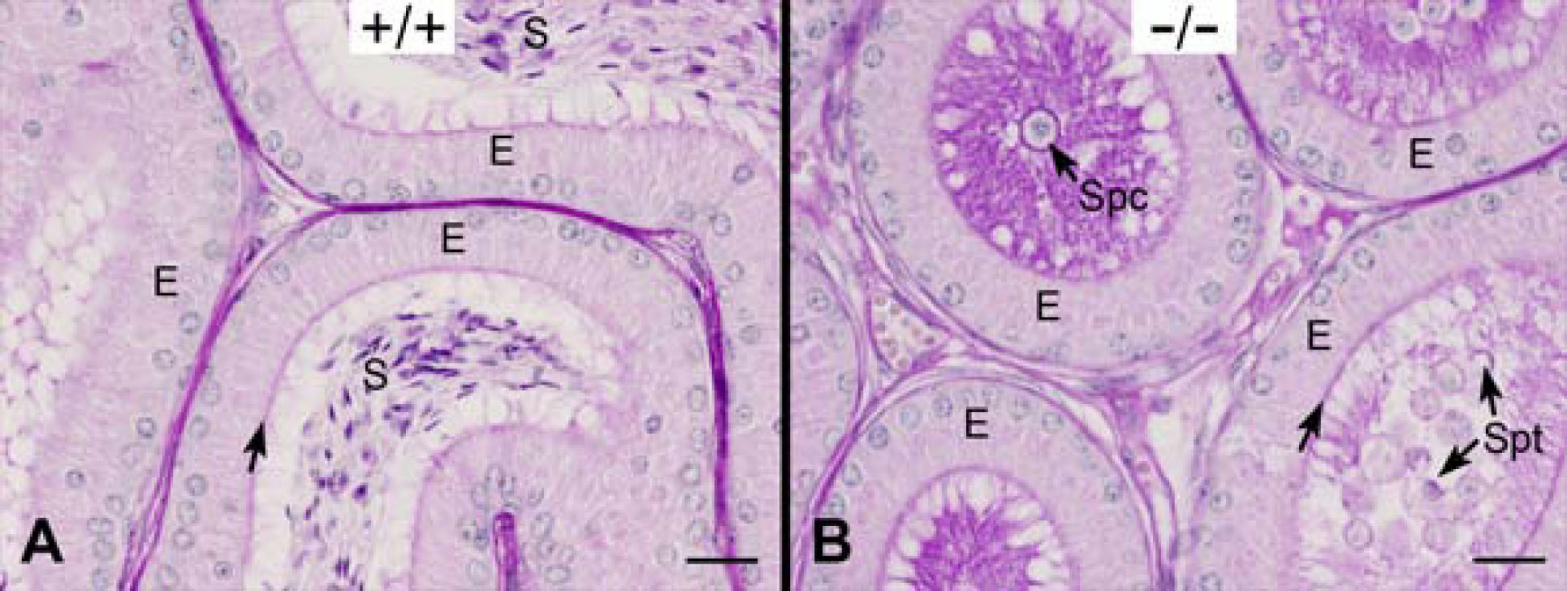
***Ehd1*^-/- ^male mice lack mature epididymal spermatozoa at day 42**. Day 42 caput epididymides were Bouin's-fixed, PAS-stained and hematoxylin-counter-stained to visualize the glycoproteins (pink) and nuclei (blue) and analyzed by light microscopy using a 40× objective lens. (A) WT (+/+) epididymides contained a columnar epithelial layer (E) with a smooth actin layer (arrow) beneath the long PAS-positive microvilli that extend into the lumen. Mature spermatozoa (S) were present in the lumen. (B) The *Ehd1*^-/- ^(-/-) epididymides contained a few sloughed round spermatids (Spt) and spermatocytes (Spc). Scale bar = 20 μm.

**Figure 9:**
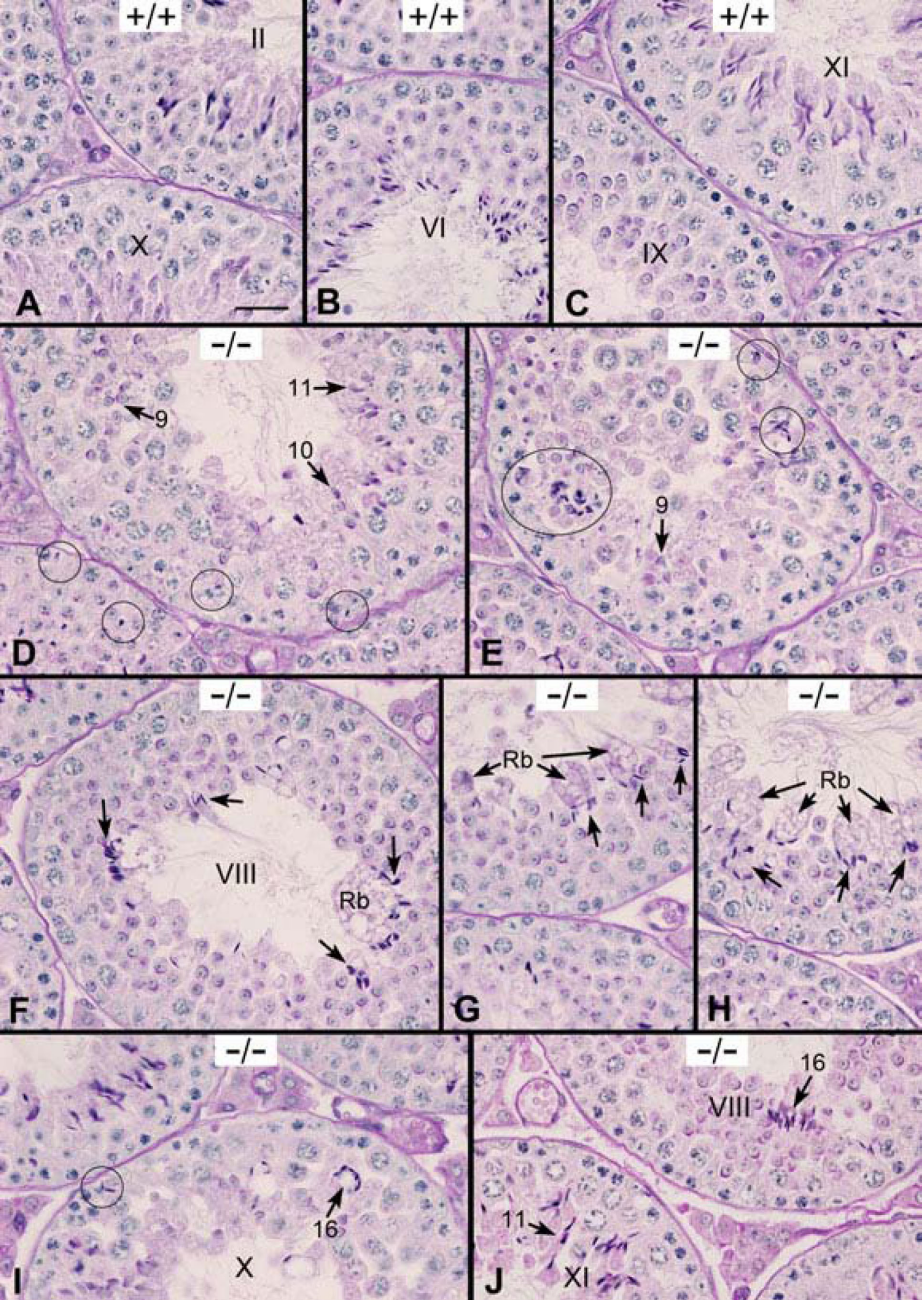
**Day 42 *Ehd1*^-/- ^testis display a delay in spermatid development and abnormal spermatid clumping**. Day 42 testes were Bouin's-fixed, PAS-stained and hematoxylin-counter-stained to visualize the glycoproteins/acrosomes (pink) and nuclei (blue) and analyzed by light microscopy using a 40× objective lens. Stages are labeled with Roman numerals. (AC) WT (+/+) seminiferous tubules displayed evenly spaced round and elongated spermatids. (D) *Ehd1*^-/- ^(-/-) seminiferous tubules contained step 9, 10, and 11 elongated spermatids (arrows) and misoriented step 16 elongated spermatids near the basement membrane (circles) and (E) abnormal step 9 spermatids along with aggregates of step 16 spermatids (circles). (F-H) *Ehd1*^-/- ^seminiferous tubules displayed membranous wheels or residual bodies (arrows, Rb) containing clumped spermatids near the lumen in stage VIII, (I) misaligned spermatids (circle) and clumped step 16 spermatid nuclei (arrows) in stage X, (J) abnormal step 11 spermatids in stage XI and step 16 spermatids clumping in membranous wheels in stage VIII. Scale bar = 50 μm.

At day 42, the epididymides in WT mice contained mature spermatozoa whereas *Ehd1*^-/- ^mice lacked sperm (Figure 8); this defect continued into adulthood. To gain further insights into abnormal spermatogenesis, we carried out a detailed examination of 42 day old WT and *Ehd1*^-/- ^mouse testes. WT mice displayed normal spermatogenesis where a single step of round and elongated spermatids were supported by Sertoli cells in an evenly spaced and orderly fashion in seminiferous tubule cross-sections (Figure 9A-C). On the other hand, spermatogenesis only appeared normal prior to acrosome formation in *Ehd1*^-/- ^mice. Several *Ehd1*^-/- ^seminiferous tubule cross-sections exhibited a mixture of elongated spermatids (Figure 9D, steps 9-11) as well as misaligned elongated spermatids near the basement membrane, suggesting Sertoli cell phagocytosis of step 16 spermatids that failed to be released (Figure 9D-E, circles). In late stage VIII, failure of spermiation and clumping of spermatid heads was observed in addition to fusion of large aggregates of residual bodies and cytoplasmic lobes that contained clumped spermatids (Figure 9F-H, arrows). In stage X, clumping of step 16 spermatids was observed in membranous wheels and near the basement membrane (Figure 9I). Step 16 spermatids were also observed with their heads and tails fused; their cytoplasm failed to form cytoplasmic lobes and residual bodies which are normally reabsorbed by Sertoli cells (Figure 9J). *Ehd1*^-/- ^testis also showed abnormal step 11 spermatids (Figure 9J). Thus, our results demonstrate clear spermatogenesis and spermiation defects in *Ehd1*-null testes.

**Figure 10:**
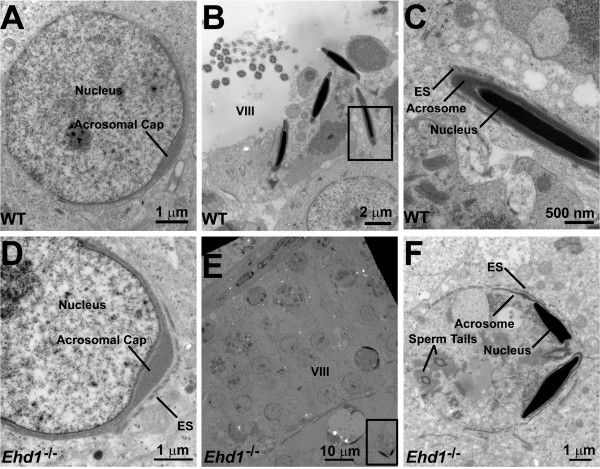
**Electron micrographs of day 45 seminiferous tubules reveal abnormal phagocytic membranous wheels in *Ehd1*^-/- ^mice**. (A) In WT mice, step 8 round spermatids were found in (B) early stage VIII seminiferous tubules that contained elongated spermatids in the process of spermiation near the lumen with tails apparent after spermiation; box enlarged in (C). (C) A WT elongated spermatid prior to spermiation maintains its ectoplasmic specializations (ES). (D) In *Ehd1*^-/- ^mice, step 8 round spermatids were found in (E) late stage VIII seminiferous tubules that contained phagocytic membranous wheels engulfing elongated spermatids, their acrosomes and sperm tails; box enlarged in (F).

To further characterize the defects in spermatogenesis in *Ehd1*^-/- ^mice at the ultrastructural level, transmission electron microscopy analyses were carried out on thin sections of the testis. In stage VIII of WT testis (Figure 10A), elongated spermatids were found near the lumen or in the lumen after spermiation (Figure 10B). Elongated spermatids that had not spermiated maintained an apical ectoplasmic specialization in contact with WT Sertoli cells (Figure 10C). However, in late stage VIII of *Ehd1*^-/- ^testis (Figure 10D), elongated spermatids appeared in phagocytic, membranous wheels (Figure 10E, box). Upon closer examination, the phagocytic wheel was encased by ectoplasmic specializations and contained the nuclei, acrosomes and tails of elongated spermatids (Figure 10F). Since proper function of the Sertoli cells requires constant endocytic trafficking [31], we surmise that EHD1-dependent endocytic recycling and trafficking may be required for spermiation in mice.

## Discussion

An earlier report of an *Ehd1 *knockout mouse indicated no overt biological phenotype [27] which was surprising given the *in vitro *cell biological evidence that supports the critical roles of EHD proteins in endocytic recycling. Here, we show that mice that completely lack EHD1 expression exhibit multiple overt phenotypes, consistent with a critical role of EHD1 during pre- and post-natal development and organ function. While reduction of EHD1 in adult *Ehd1*^+/- ^mice was without detectable biological phenotypes, *Ehd1*^-/- ^mice survived at sub-Mendelian ratios and exhibited reduced body size. *Ehd1*^-/- ^mice also showed higher early post-natal mortality and exhibited two dramatic organ-specific phenotypes: marked abnormalities in eye development (which is not pursued further here) and male infertility (which is described in detail here).

Spermatogenesis is a complex process in which diploid spermatogonia develop into mature haploid spermatozoa capable of fertilizing an ovum. In the testis, somatic Sertoli cells nurse as many as ~30-50 developing germ cells [28]. During maturation, developing spermatids remain attached to Sertoli cells through specialized membrane structures. The final junction between spermatozoa and the Sertoli cell is an actin-based testis-specific adherens junction termed the apical ectoplasmic specialization that forms in late stage VII and early stage VIII of spermatogenesis [32,33]. Spermiation occurs at late stage VIII when polarized spermatozoa are released from Sertoli cells and enter the lumen.

The observed defects in spermatogenesis in *Ehd1*^-/- ^male mice can explain their infertility. First, mature spermatozoa are not found in the epididymides of *Ehd1*^-/- ^males. Second, this is a direct result of the failure of spermiation. Third, spermiation failure can be explained by abnormal clumping of elongated spermatids in membranous wheels followed by phagocytosis by Sertoli cells in stages VIII-X. Fourth, a delay in the progression of spermatogenesis results in a mixture of spermatid steps. Fifth, abnormal formation of the acrosomal granule and cap of round spermatids was the earliest lesion observed. Since only minor abnormalities were detected in day 10 testes, these results implicate EHD1 in pre-pubertal development of the testis and suggest that potential EHD1-dependent endocytic recycling mechanisms in the haploid phase of spermatogenesis may be required for normal acrosome development and spermiation. Ultrastructural analyses of *Ehd1*^-/- ^mouse testes further support these data.

Abnormal spermatogenesis and male sterility in *Ehd1*^-/- ^mice indicate that either EHD2, EHD3 and EHD4, are insufficient to compensate for the loss of EHD1 or that EHD1 is uniquely important. Recent immunohistochemical analysis suggested that EHD1 is particularly highly expressed in elongated spermatids [27] while our data suggests that Sertoli cells and spermatogonia also express EHD1 (Figure 5B-C, panel A). Western blot analyses of an immortalized mouse Sertoli cell line also indicated that EHD1, EHD2 and EHD4 proteins are expressed in Sertoli cells (Figure 5A, lower panel) with lower/undetectable levels of EHD3. Availability of knockout models lacking the expression of the other EHD family members (currently under development in the laboratory) should help to unravel the redundant versus unique EHD1 functions in spermatogenesis. Analyses of *Ehd4*-null mice, in which both males and females are fertile but male testis sizes are ~50% smaller than WT mice at day 31, indicate that EHD4 is required for mice to attain normal pre-pubertal testis size but is dispensable for male fertility (George *et al.*, manuscript accepted for publication).

How might the loss of endocytic recycling regulator EHD1 lead to a block in spermatogenesis and spermiation? The data described here indicate defects in spermiogenesis, a process where major restructuring of spermatids occurs after the blood-testis barrier has formed at the tight junctions of Sertoli cells. Germ cell differentiation is coordinated by Sertoli cells and the ectoplasmic specializations formed between Sertoli and maturing germ cells in the adult animal. Once established, these Sertoli-germ cell complexes move with germ cells until spermiation occurs [34]. In *Ehd1*^-/- ^mice, the acrosomal cap that associates with the junctional complexes appear to develop abnormally. This could affect the ability of Sertoli cells to regulate germ cell migration and thus result in clumping of spermatids and failure of release. EHD1 expression in germ cells and Sertoli cells indicate the abnormalities observed could arise from defects in either cell type or both.

Endocytosis and recycling of integral membrane proteins has recently emerged as an important regulator of spermatogenesis [31]. Since EHD proteins are known to regulate endocytic recycling in other systems, we hypothesize a role for EHD1 in endocytic recycling during spermatogenesis. Synchronous regulation of the actin-based ectoplasmic specializations and components of the blood-testis barrier (gap junctions, adherens junctions and tight junctions) co-ordinate spermatogenesis [33,35]; in addition, these dynamic structures are regulated by hormones, growth factors and cytokines to precisely control the timing of the passage of developing germ cells via endocytic recycling mechanisms [31,36]. Indeed, cytokines implicated in transient physiological opening of the blood-testis barrier have been shown to induce the internalization of Sertoli cell junctional proteins [36,37]. Notably, IL-1, a cytokine implicated in the regulation of Sertoli-Sertoli cell junctions [38], has been shown to up-regulate the expression of *Ehd1 *mRNA in other model cell systems [39]. How Sertoli cell and/or germ cell membrane proteins involved in regulating ectoplasmic specializations are recycled back to the cell membrane is completely unknown. In this context, detailed future studies of the trafficking of gap junction-, tight junction- and adherens junction-associated proteins in the WT and *Ehd1*^-/- ^testis as germ cells migrate from the basement membrane into the adluminal compartment should provide a better understanding of EHD1-regulated processes during spermatogenesis. Analyses of Sertoli-Sertoli and Sertoli-germ cell interactions *in vitro *combined with Sertoli vs. germ cell-specific EHD1 knockout should further help test these models and ascertain the biochemical and cell biological processes in spermatogenesis that are under EHD1 regulation.

What might lead to the small testes phenotype in the *Ehd1*^-/- ^mice? Since elongated spermatids are phagocytosed rather than spermiated in the *Ehd1*^-/- ^testes, there are less germ cells in the seminiferous epithelium. The presence of Sertoli cell-only seminiferous tubules in the *Ehd1*^-/- ^testes indicates that progressive loss of germ cells might occur and we have observed enhanced apoptosis in the *Ehd1*^-/- ^testes (data not shown). Since the majority of adult testis weight is due to the presence of germ cells in the seminiferous epithelium [40], germ cell depletion due to phagocytosis, defects in differentiation or increased apoptosis could be responsible for the small testis phenotype.

At present, the developmental defects and growth retardation seen in *Ehd1*^-/- ^mice is under investigation. Our results contrast with the lack of observable phenotypes in the EHD1 knockout mice previously described [27]. The differences in strains (129Sv/Ev or Swiss Webster versus mixed 129;B6) or the strategies used to delete *Ehd1 *(part of exon 3 and 5 and all of exon 4 versus exon 1 in our studies) could have led to the differences observed. Whether the lack of a phenotype of EHD1 deletion in the previous studies was a result of full compensation by other EHD proteins or due to expression of a truncated but functional EHD1 is not known. An increase in the expression of other EHD proteins is seen in the day 30-42 *Ehd1*^-/- ^testes described here (Figure 5A, upper panel), yet the presence of fertility defects in the *Ehd1*^-/- ^mice indicate that this does not compensate for loss of EHD1. In order to ensure that N-terminally-truncated in-frame fragments of EHD1 were not present in *Ehd1*^-/- ^mice, Western blots of organ lysates were probed with an EHD1 antibody that was raised against the C-terminal region. As expected, we did not observe the ~61 kD full length EHD1 in *Ehd1*^-/- ^mouse organs including the testis (Figure 2, Figure 5A). RT-PCR with two primer sets specific to the C-terminal region of *Ehd1 *showed no amplification of products using mRNA isolated from *Ehd1*^-/- ^mouse testes when compared to expected products with WT testes (Figure 1C). Furthermore, *Ehd1*^*fl-Neo/fl-Neo *^mice with *loxP *sites flanking the first exon of EHD1 were normal with respect to development, growth and fertility. These results indicate that specific loss of EHD1expression is responsible for the defects described.

## Conclusions

Our analyses using an *Ehd1*^-/- ^mouse model with complete loss of EHD1 expression demonstrates an important role of this novel regulator of endocytic recycling in mammalian development with a critical functional role in spermatogenesis. This model provides a basis for further studies to explore the physiological targets of EHD1 and the biological processes regulated by this protein as well as to explore how endocytic recycling controls spermatogenesis. Thus our results indicate for the first time, a crucial role of an endocytic recycling regulatory protein EHD1 in spermatogenesis and provide the first evidence of a critical *in vivo *biological function of a mammalian EHD protein family member.

## Methods

### Generation of *Ehd1 *gene-targeted mice

A conditional gene knockout targeting vector was generated using the "recombineering" method [41]. In brief, we identified a BAC clone RPCI-22-373M7 containing the mouse *Ehd1 *gene from the RPCI-22 mouse (129S6/SvEvTac strain) BAC library high-density filters (Children's Hospital Oakland Research Institute, http://bacpac.chori.org). Using a series of "recombineering" reactions, an ~11.7 kb fragment of the BAC DNA containing the first and second exons of *Ehd1 *was retrieved into a plasmid. Two *loxP *sites were introduced flanking exon 1. The second *loxP *site was immediately preceded by an engineered *FRT-Neo-FRT *selection cassette that conferred G418 resistance in transfected ES cells. The *Neo *gene could be removed from the gene locus with the expression of FLP DNA recombinase, leaving behind single *FRT *and *loxP *sequences thereby keeping the alterations of the gene locus to a minimum. The recombineering reagents (plasmids and bacterial strains) were obtained from Dr. Neal G. Copeland at the National Cancer Institute, Frederick, Maryland. PCR primer sequences used to generate the targeting vector as well as to generate probes for Southern hybridization are listed (see Additional file 1).

Homologous recombination was carried out in mouse embryonic stem cells to generate a targeted *Ehd1 *allele using *loxP *sites flanking exon 1 of the *Ehd1 *gene. The targeting vector was linearized with *Not*I and electroporated into HM1, an ES cell line derived from the 129/Ola mouse strain. We screened 95 clones after G418 and gancyclovir selection by Southern hybridization using 5' and 3' external probes and identified 6 correctly-targeted clones. Two correctly-targeted ES cell clones were injected into C57BL/6J blastocysts to yield chimeric mice. One achieved germline transmission of the targeted *Ehd1 *allele. Chimeric mice were mated with C57BL/6J mice and those with germline transmission of a targeted allele (*Ehd1*^*flox*/+^) were selected.

*Ehd1*^*flox*/+ ^mice were mated with B6.FVB-Tg(EIIa-Cre)C5379Lmgd/J mice expressing Cre recombinase from the adenovirus EIIa promoter to generate heterozygote mice (*Ehd1*^+/-^). *Ehd1*^+/-^;*cre *transgene-positive mice were crossed to C57BL/6J mice to generate heterozygous *Ehd1*-deleted, *cre *transgene-negative (*Ehd1*^+/-^) mice, which were subsequently used to produce EHD1-deficient mice (*Ehd1*^-/-^). Alternatively, *Ehd1*^*flox*/+ ^mice were mated with B6;SJL-Tg(ACTFLPe)9205Dym/J mice expressing the enhanced *FLP1 *recombinase (FLPe) from the human beta Actin (*ACTB*) promoter to remove the *FRT*-flanked *Neo *gene. *Ehd1*^*flox*/+^;*FLPe *transgene-positive mice were crossed to C57BL/6J mice to generate heterozygous *Ehd1*-floxed, *FLPe *transgene-negative (*Ehd1*^*fl-Neo*/+^) mice. Crosses of *Ehd1*^*fl-Neo*/+^mice gave rise to homozygous floxed mice (*Ehd1*^*fl-Neo/fl-Neo*^). These mice were used in some experiments to confirm that the observed phenotypes were due to loss of EHD1 expression and not due to insidious genetic aberrations associated with the ES cell clone used to generate the *Ehd1 *mutant mice. All mice were purchased from The Jackson Laboratory.

### Genotyping

Mouse tail DNA was extracted according to protocol (Gentra Puregene Mouse Tail Kit, Qiagen catalog #158267) and hydrated in water. PCR analysis of DNA was carried out using three primers (primers1-3) in a duplex PCR reaction as described (see Additional file 2). PCR products were separated using 2% agarose gel electrophoresis to determine the genotypes with various *Ehd1 *alleles.

### Reverse transcriptase polymerase chain reaction (RT-PCR)

Following euthanasia of mice, the testes were dissected from the scrotal sac and flash-frozen in liquid nitrogen. Total RNA was isolated according to the TRI Reagent RT protocol (Molecular Research Center, Inc.) with substitution of chloroform for 4-bromoanisole during the phase separation. RNA (1 μg) was denatured at 65°C for 5 min in the presence of 0.5 μg oligo(dT)_15 _primer (Promega, Madison, WI) and 0.83 mM dNTPs (New England Biolabs, Ipswich, MA) in a total volume of 12 μL of DEPC-treated water followed by quick annealing on ice. The total reaction volume for reverse transcription was brought to 20 μL reaction using 1× first strand buffer (Invitrogen), 10 mM dithiothreitol (Invitrogen) and 50 units of Moloney murine leukemia virus reverse transcriptase (Stratagene, La Jolla, CA) in DEPC-treated water and placed at 42°C for 50 min. The reverse transcriptase was heat-inactivated at 70°C for 10 min. The resulting first strand cDNA was used as a template in a PCR reaction as described (see Additional file 2) to amplify a 394 bp product of exons 4 and 5 of *Ehd1 *using previously described primers (termed primersA here) [27]; primers 4 and 5 (see Additional file 2) to amplify a 261 bp product of exons 3 and 4 of *Ehd1 *(termed primersB); and the 3' untranslated region of *Ehd4 *was amplified using previously described primers [27] to yield a 342 bp product (termed primersC here).

### Animal husbandry and care

All experiments involving animals were approved by the Institutional Animal Care and Use Committee and were treated humanely in accordance with the institutional guidelines and those in the National Institutes of Health (NIH) Guide for the Care and Use of Laboratory Animals. For most studies, breeding of *Ehd1*^+/- ^mice was used to generate WT (*Ehd1*^+/+^), *Ehd1*^+/- ^and *Ehd1*^-/- ^mice. In some cases, *Ehd1*^-/- ^females were bred with *Ehd1*^+/- ^males to generate *Ehd1*^+/- ^and *Ehd1*^-/- ^mice. For all breeding studies, *Ehd1*^-/- ^male mice (8-weeks of age) were housed with two females for eight weeks to determine fertility.

### Antibodies and Western blotting

Previously described rabbit anti-peptide antibodies against human EHD proteins were utilized [7]. Similarly generated antibodies against a synthetic EHD1 peptide (amino acids 519-534: CADLPPHLVPPSKRRHE) was cross-reactive with EHD1 and EHD4 and was used either without purification to immuno-blot EHD1 and EHD4 (Figure 2, Figure 5A, lower panel), or was Protein G- purified for immuno-blotting (Figure 5A, upper panel) and immunohistochemistry (Figure 5B-C). The affinity purified antibodies preferentially recognize EHD1 with low reactivity against EHD4 (Figure 5A, upper panel). Tissue and cell lysates were prepared and immuno-blotted as described [7], using 20-100 μg lysate protein aliquots, primary antibodies at 1:2000 and Protein A-HRP conjugate (Invitrogen, #10-123) at 1:20,000 dilution. In Figure 2, the membrane was serially stripped and reprobed beginning with antisera that recognize EHD1 and EHD4, followed by EHD2, EHD3 and Hsc70 antibodies; blots shown have exposure times of less than 10 seconds, upon longer exposures, most EHD proteins can be seen in each organ shown. In Figure 5A, upper panel, blots shown have exposure times of 3 min for EHD1, EHD2 and EHD4; exposure of 10 min for EHD3. TM4 cells were obtained from ATCC (#CRL-1715). *Ehd1*^*fl-Neo/fl-Neo *^mouse embryonic fibroblasts were obtained in-house using standard protocols [42].

### Testis preparation and staging

Animals were euthanized, the testes and epididymides were removed, weighed and immediately immersed in Bouin's fixative overnight. The fixed samples were extensively rinsed in water, stored in 50% ethanol overnight and transferred to 70% ethanol overnight prior to embedding in paraffin. Transverse sections (5 μm) were prepared on glass slides, deparaffinized, and stained with Periodic Acid-Schiff (PAS)-based stain and hematoxylin as a counter-stain according to the manufacturer's protocol (Sigma-Aldrich, St. Louis, MO, #395B). Staging of seminiferous tubules was performed on light microscopy images.

### Immunohistochemistry

Freshly removed testes were poked through the capsule once at each pole with a 25 gauge, 5/8" needle to facilitate diffusion of the fixative, immersed overnight in 10% neutral buffered formalin and transferred to 70% ethanol prior to paraffin embedding. Transverse sections (5 μm) were deparaffinized in xylene and rehydrated in graded ethanols followed by PBS. For antigen retrieval, the slides were boiled twice for 10 min in citrate-based antigen unmasking solution (Vector Laboratories, Burlingame, CA, #H-3300) in a microwave. Endogenous peroxidase was inactivated by a 15 min incubation in 3% hydrogen peroxide (Sigma-Aldrich, St. Louis, MO) in PBS. Staining was carried out using the Zymed Laboratories Histostain-SP Kit (Broad Spectrum, DAB, Invitrogen, Carlsbad, CA, #95-9643). The affinity purified rabbit-anti-EHD1 primary antibodies were used at a 1:250 dilution in PBS/5% fetal bovine serum.

### *In situ *hybridization

PCR amplification of the 3' UTR of *Ehd1 *(nucleotides 1741-2220 [GenBank:NM_010119]) from WT mouse testis cDNA was carried out and cloned into pCR4-TOPO (Invitrogen) and sequenced. To analyze *Ehd1 *mRNA expression, [^35^S] UTP-labeled riboprobes were generated. The *Ehd1 *antisense riboprobe was synthesized using *Pst*I-digested *Ehd1 *DNA and T7 RNA polymerase (Promega, Madison, WI). *In situ *hybridizations were performed using the same hybridization and washing conditions as described previously [43] on 10% neutral buffered formalin-fixed testis sections that were paraffin-embedded and mounted on StarFrost glass slides (Mercedes Medical, Sarasota, FL). The hybridized slides were soaked in Kodak NTB-2 emulsion, dried and exposed for 8-10 days at 4°C. Following development and fixation, the slides were counter-stained with hematoxylin. Bright- and dark-field images were captured separately using a Nikon Eclipse E600 microscope. Silver grains in the dark-field images were pseudo-colored red using ADOBE Photoshop CS and overlaid on corresponding bright-field images.

### Serum testosterone measurements

Blood was collected from euthanized animals using cardiac puncture and allowed to clot. Serum testosterone levels were measured using a radioimmunoassay at the University of Virginia Center for Research in Reproduction Ligand Assay and Analysis Core.

### Electron microscopy

Freshly removed testes were poked through the capsule ten times at equidistant sites with a 25 gauge, 5/8" needle to facilitate diffusion of the fixative, immersed overnight in 0.1 M Sorensen's phosphate buffer containing 2% glutaraldehyde and 2% paraformaldehyde, washed in 0.1 M Sorensen's phosphate buffer, post-fixed in 1% OsO_4 _aqueous solution, washed in distilled water, dehydrated in a series of ethanol followed by propylene oxide and embedded in Araldite. Sagittal cross-sections of 60-90 nm thick were placed on 200 mesh uncoated copper grids, stained with 2% uranyl acetate aqueous and Reynold's lead citrate and examined on a Philips 410LS transmission electron microscope operated at 80 kV. Digital images were recorded with an AMT digital imaging system. Reagents were obtained from Electron Microscopy Sciences.

## Abbreviations

EEs: early endosomes; EH: Eps15 homology; EHD: C-terminal Eps15 homology domain-containing protein; ERC: endocytic recycling compartment; MHC: major histocompatibility complex; RME-1: Receptor-Mediated Endocytosis-1; PAS: Periodic Acid-Schiff.

## Authors' contributions

MAR designed the study, maintained the mouse colony, acquired animal data, performed RT-PCR, organ lysis, cell culture, Western blots, antibody purification, immunohistochemistry, assisted with the testis histology, analyzed the electron microscopy data and drafted the manuscript; MG helped design the study, assisted with the testis histology, analyzed the electron microscopy data and helped draft the manuscript; MG and GY characterized the anti-EHD antibodies; RA carried out molecular biology for generation of the mouse model; DJB, ES and VG performed *in situ *hybridizations; TB and GLT made thin sections and performed the electron microscopy; LD performed blastocyst injections; SEC performed the initial mouse phenotyping; RAH performed the histopathological analysis, prepared the histology figures and helped in drafting the manuscript; MN designed and derived the mouse model, and edited the manuscript; VB and HB conceived the mouse model and secured support for the work; HB led the project, arranged collaborations and edited the manuscript. All authors read and approved the final manuscript.

## Supplementary Material

Additional file 1 **Primers for generating the *Ehd1 *targeting construct and probes for Southern hybridization**. A table containing mouse primers. Click here for file

Additional file 2 **Primers for genotyping WT, *Ehd1*^+/-^, *Ehd1*^-/-^, *Ehd1*^*fl-Neo*/+ ^and *Ehd1*^*fl-Neo/fl-Neo *^mice and amplification of *Ehd1 *cDNA**. A table containing mouse primers for PCR and RT-PCR. Click here for file
