# Supplementary material for: The endocytic recycling regulator EHD1 is essential for spermatogenesis and male fertility in mice
Source: BMC Dev Biol. 2010 Apr 2;10:37. doi: 10.1186/1471-213X-10-37 (PMC2856533; doi:10.1186/1471-213X-10-37)
Supplement: Additional file 1 — Primers for generating the Ehd1 targeting construct and probes for Southern hybridization. A table containing mouse primers. [file 1471-213X-10-37-S1.PDF]

Additional file 1 - Primers for generating the *Ehd1* targeting construct and probes for Southern hybridization

Primer	Sequence
mEHD1-A-NotI	5’-ATAAGCGGCCGCCTCAGCCTCTCGAGGCACAGGCAC-3’
mEHD1-B	5’-GCTGGTTGGATTCTTGGTTGTTCTC-3’
mEHD1-Y-SpeI	5’-TCTACTAGTCAGGCTCACCCACTCTTGATGAGT-3’
mEHD1-Z-XbaI	5’-TGCTCTAGACTTTACAGGCGCAAGCTTGTGTGG-3’
mEHD1-SalI	5’-ATGCGTCGACACTGGGCTTGCCTGAGGCTCCTGTCAC-3’
mEHD1-EcoRI	5’-CCGGAATTCTTTCTCCTCCATATGTTCCACCTTCT-3’
mEHD1-E-BamHind	5’-CGCGGATCCAAGCTTTGAGAGAGGAGGAGGTGGGAGATTTGC-3’
mEHD1-F-NotI	5’-ATTTGCGGCCGCTACCATGGTGGAGGGGGTGAGTGTGCC-3’
mEHD1-G-SalI	5’-ATGCGTCGACCCTCAGGAGAGACTGGAAGACTTGCTG-3’
mEHD1-H-EcoRI	5’-CCGGAATTCCTGTACCCCAGGCCAGACCTCCTAAGT-3’
mEHD1-I-BamHI	5’-CGCGGATCCAAGAACTATACATCCATTTCCTT-3’
mEHD1-J-NotI	5’-ATTTGCGGCCGCACAGGGATGGGGAGTTGAGTCCTTAGG-3’
EHD1K	5’-GGGCTACCTTGCAGAACTGAATGGAA-3’
EHD1L	5’-CACTCCCTCCCCTCATTCCTTCAGAG-3’
EHD1M	5’-TTAAGTCCATGGAGAGGCAGCACGGT-3’
EHD1N	5’-AGGGTTACCCTTGGCTACCTATGAAG-3’
EHD1O	5’-GAGCTCTGCTGTTTGTGCCGCCTTCG-3’
EHD1P	5’-TTGAATTTCCTGCACATGAGGGACCA-3’
EHD1Q	5’-GTTCCTGAGTGCTACCCGTGTGACTG-3’
EHD1R	5’-AAGCTACTCAGGGTGTAGCCCTCCTT-3’

The primers (mEHD1-A through mEHD1-J) were used to generate the *Ehd1* targeting construct. The primer nomenclature (A, B, Y, Z, etc.) is according to Liu et al. [41]. In order to generate probes for Southern hybridization, PCR was performed using the primers (EHD1K through EHD1R) and BAC DNA as a template. PCR amplicons generated using K and L primers and M

and N primers were mixed and used as the 5’ probes. PCR amplicons generated using O and P primers and Q and R primers were mixed and used as 3’ probes.
