# Supplementary material for: The endocytic recycling regulator EHD1 is essential for spermatogenesis and male fertility in mice
Source: BMC Dev Biol. 2010 Apr 2;10:37. doi: 10.1186/1471-213X-10-37 (PMC2856533; doi:10.1186/1471-213X-10-37)
Supplement: Additional file 2 — Primers for genotyping WT, Ehd1+/-, Ehd1-/-, Ehd1fl-Neo/+ and Ehd1fl-Neo/fl-Neo mice and amplification of Ehd1 cDNA. A table containing mouse primers for PCR and RT-PCR. [file 1471-213X-10-37-S2.PDF]

Additional file 2 - Primers for genotyping WT, *Ehd1*$^{+/-}$, *Ehd1*$^{-/-}$, *Ehd1*$^{fl\text{-}Neo/+}$ and *Ehd1*$^{fl\text{-}Neo/fl\text{-}Neo}$ mice and amplification of *Ehd1* cDNA

Primer	Sequence	Comments
1	5’-AAGTCAGAAGACAACTTTCTGGAGTTCCCT-3’	*Ehd1* mice genotyping
2	5’-TCCAGGGCCCACATGGTAGAAGGAGAGAGT-3’	*Ehd1* mice genotyping
3	5’-GCTCCGGTCTTGGACTTCACCAGCATTTAG-3’	*Ehd1* mice genotyping
4	5’-TGGTCACACCCACTGCTCATC-3’	*Ehd1* cDNA amplification
5	5’-CCCGCTCGATCTTCTGGT-3’	*Ehd1* cDNA amplification

A duplex PCR reaction was used to genotype WT, *Ehd1*$^{+/-}$, *Ehd1*$^{-/-}$, *Ehd1*$^{fl\text{-}Neo/+}$, and *Ehd1*$^{fl\text{-}Neo/fl\text{-}Neo}$ mouse tail DNA using equal quantities of primers 1-3. The product of primer 1 and 2 yields a 403 bp WT band and the product of primer 2 and 3 yields a 305 bp deleted band for an *Ehd1*$^{+/-}$ mouse. The same duplex PCR reaction was utilized to genotype *Ehd1*$^{fl\text{-}Neo/+}$ and *Ehd1*$^{fl\text{-}Neo/fl\text{-}Neo}$ mice which yielded a 501 bp band (Figure 1B). An RT-PCR reaction (Figure 1C) utilized previously described primer sets to amplify mouse *Ehd1* and *Ehd4* cDNA, respectively [27] while primers 4 and 5 were used to amplify *Ehd1* cDNA to yield a 261 bp band. Thermal cycling was carried out using 94 °C for 1 min, followed by 30 cycles of denaturation (94 °C, 30s), annealing (60 °C, 30s), extension (72 °C, 30s) and a final 5 min extension at 72 °C.
